# Supplementary material for: Pigs lacking Natural Killer T cells have altered cellular responses to influenza
Source: PLoS Pathog. 2026 Apr 6;22(4):e1014094. doi: 10.1371/journal.ppat.1014094 (PMC13068344; doi:10.1371/journal.ppat.1014094)

S1 Fig

G1 (*n*=6): *CD1D−/−* vaccinated and challenged
G2 (*n*=6): *CD1D−/+* vaccinated and challenged
G3 (*n*=5): *CD1D−/−* not vaccinated and challenged
G4 (*n*=5): *CD1D−/+* not vaccinated and challenged
G5 (*n*=3): *CD1D−/+* not vaccinated, not challenged

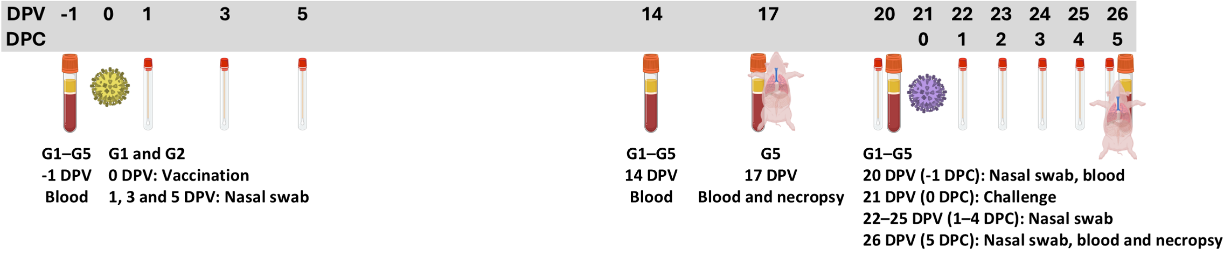

Supplement: S1 Fig — Eleven CD1D − /− and 14 CD1D − / + pigs were assigned into five groups: G1 (n = 6): CD1D − / − vaccinated and challenged; G2 (n = 6): CD1D − / + vaccinated and challenged; G3 (n = 5): CD1D − / − not vaccinated and challenged; G4 (n = 5): CD1D − / + not vaccinated and challenged; G5 (n = 3): CD1D − / + not vaccinated, not challenged. G1 and G2 were intranasally vaccinated with TX98 NS1Δ126 H3N2 at 0 day post-vaccination (DPV), while G3, G4, and G5 were left unvaccinated. At 17 DPV, control pigs in G5 were humanly euthanized for post-mortem and sample collection. G1–4 were intratracheally challenged with CO99 H3N2 at 21 DPV [0 days post-challenge (0 DPC)] and monitored for 5 days. Nasal swab and blood were collected throughout the study. Created in BioRender. Kwon, T. (2026) https://BioRender.com/htwvznn. (PDF) [file ppat.1014094.s001.pdf]
