# Supplementary figures and images for: Pigs lacking Natural Killer T cells have altered cellular responses to influenza
Source: PLoS Pathog. 2026 Apr 6;22(4):e1014094. doi: 10.1371/journal.ppat.1014094 (PMC13068344; doi:10.1371/journal.ppat.1014094)

S2 Fig

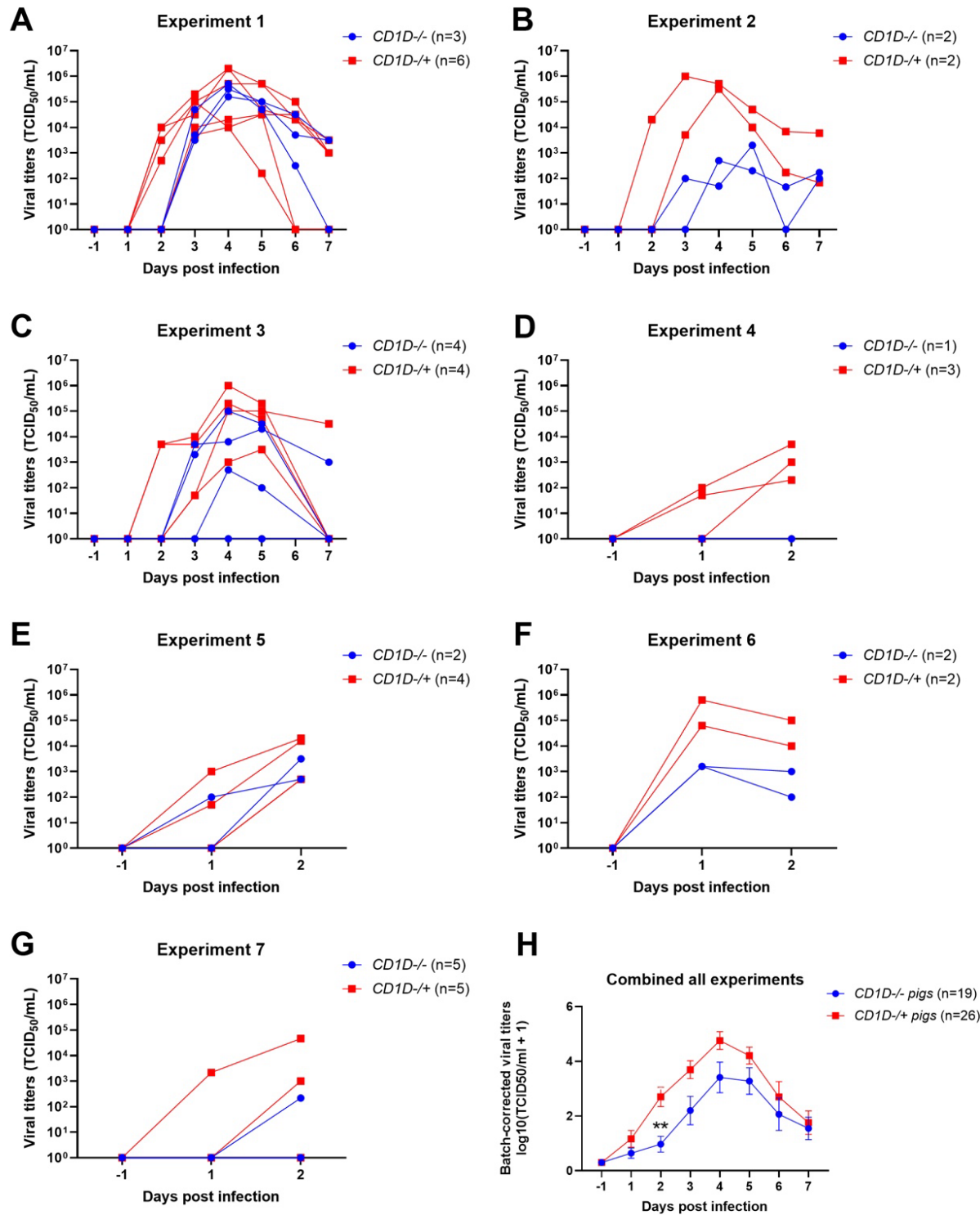

Supplement: S2 Fig — (A–G) Viral titers in nasal swabs from seven prior experiments where CD1D − /− and CD1D − / + pigs between 4 and 6 weeks of age were intratracheally infected with pandemic H1N1 A/California/04/2009 influenza A virus. Each line represents an individual pig. (H) Mean viral titers across all experiments are presented as mean ± SEM. To minimize batch effects, titers were log₁₀-transformed and normalized by centering each batch to the overall mean (adjusted value = raw − batch mean + grand mean). The adjusted values were used for statistical analyses. Treatment and time effects were assessed using a mixed-effects model (REML), followed by Sidak’s multiple comparisons test for pairwise comparisons. (PDF) [file ppat.1014094.s002.pdf]

S3 Fig

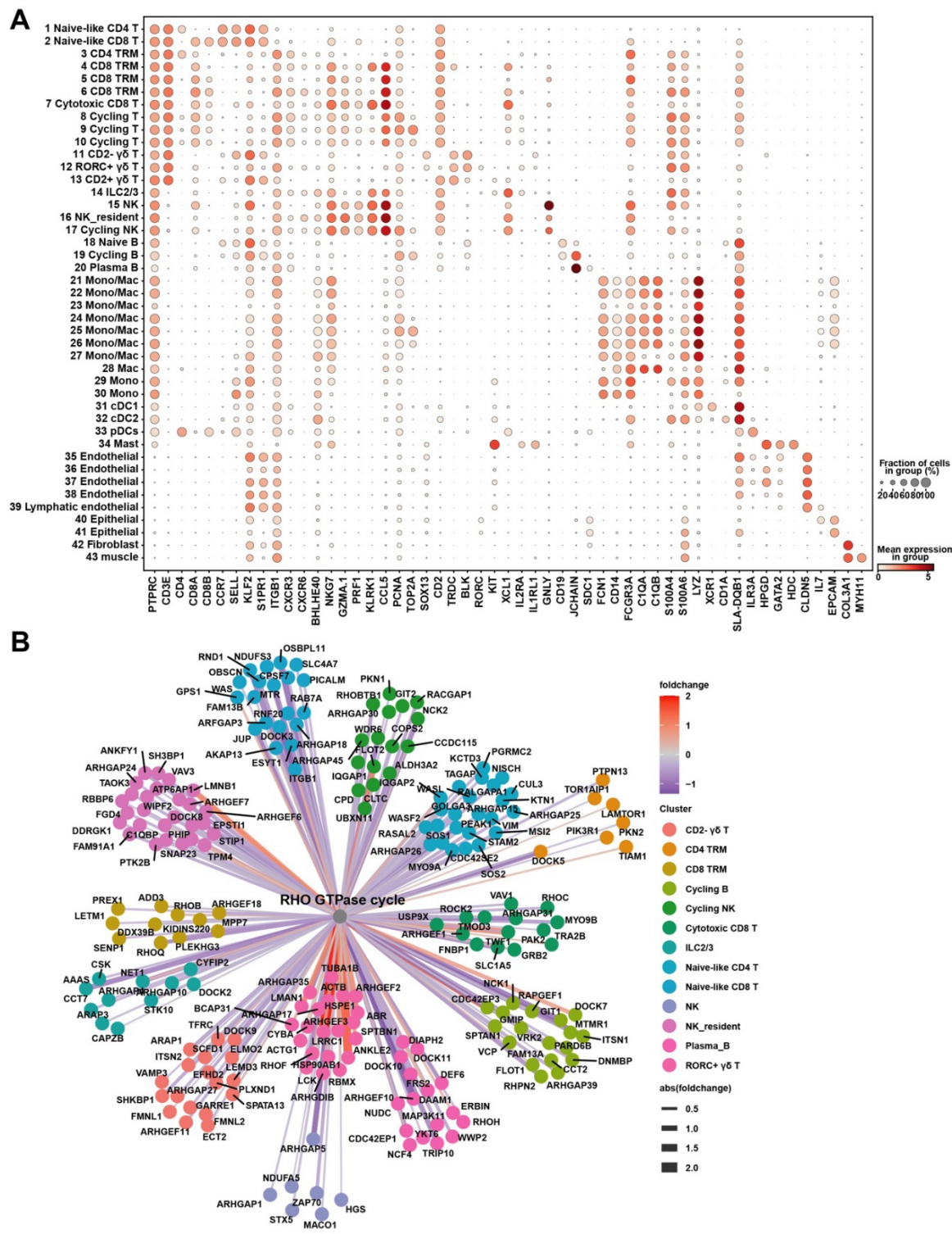

Supplement: S3 Fig — (A) Dot plot showing the mean expression of selected marker genes in each cluster from Fig 6A. (B) Network graph showing cluster-specific DEGs involved in the RHO GTPase cycle pathway. Edge color indicates positive or negative fold change in G1 compared to G2, and edge thickness represents the absolute fold change. G1: CD1D − / − vaccinated and challenged; G2: CD1D − / + vaccinated and challenged. (PDF) [file ppat.1014094.s003.pdf]

S5 Fig

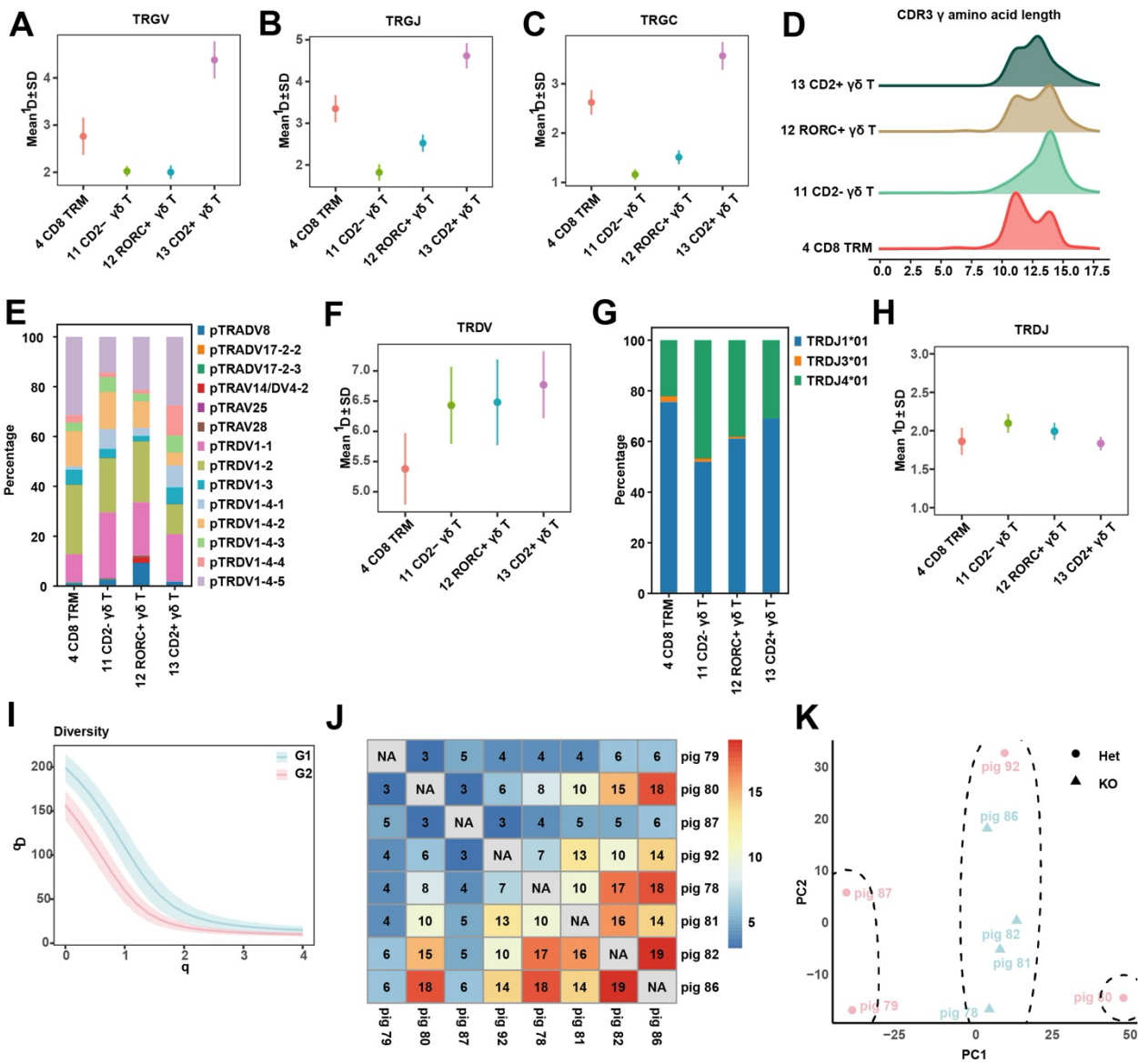

Supplement: S5 Fig — (A–C, F, H) VJC segment diversity measured using the Shannon–Wiener index, corresponding to the Hill diversity index at order q = 1 for each γδ T cell subtype. (D) CDR3γ amino acid length distribution across γδ T cell subtypes. (E, G) Proportion of cells expressing TRDV (E) and TRDJ (G) segments by cell type. (I) CDR3γ diversity across varying Hill diversity orders. (J) Heatmaps showing the number of overlapping CDR3γ sequences across samples. (K) Principal component analysis (PCA) of TRG and TRD VJC segment usages by sample. (PDF) [file ppat.1014094.s005.pdf]

S6 Fig

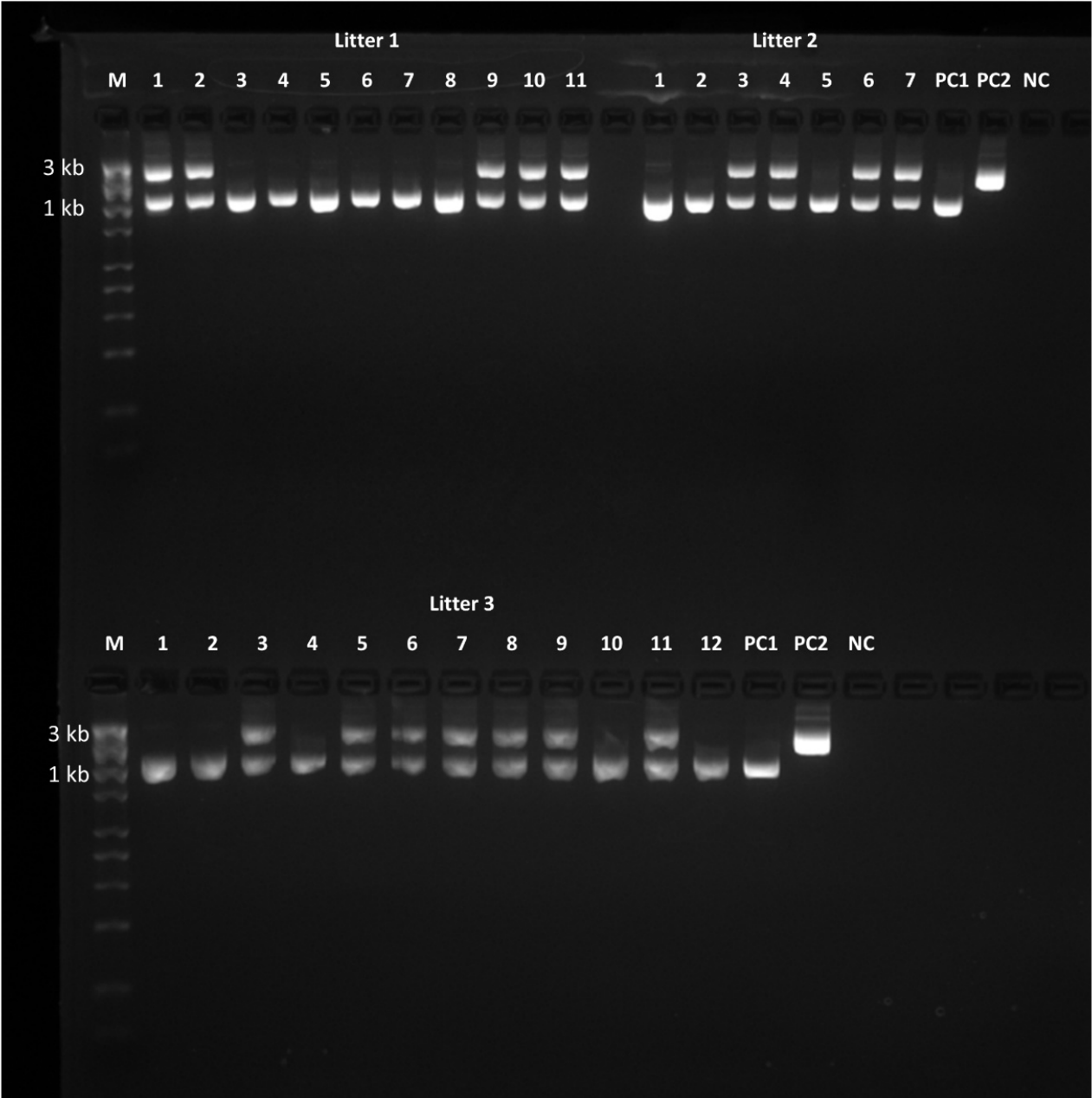

Supplement: S6 Fig — CD1D genotype of pigs from three litters was confirmed by PCR targeting a 2,788 bp product of the endogenous porcine CD1D gene. Pigs that possessed an edited CD1D allele produced a deletion of 1,598 bp resulting in a modified product of 1,189 bp. A single 1,189 bp band was detected in homozygous (CD1D − /−) pigs, whereas two PCR products at 1,189 and 2,787 bp were detected in heterozygous (CD1D − /+) pigs. M: DNA molecular marker; PC1: a positive control for the modified allele; PC2: a positive control for an unmodified, “wildtype” sample; NC: negative control. Pigs #1–11 (1–6 are female and 7–11 are male) from litter 1, #1–7 (1–4 are female and 5–7 are male) from litter 2, and 1, 3 and 6–10 (1 and 3 are female and 6–10 are male) from litter 3 were used in this study. (PDF) [file ppat.1014094.s006.pdf]
