# Supplementary material for: Pigs lacking Natural Killer T cells have altered cellular responses to influenza
Source: PLoS Pathog. 2026 Apr 6;22(4):e1014094. doi: 10.1371/journal.ppat.1014094 (PMC13068344; doi:10.1371/journal.ppat.1014094)
Supplement: S4 Fig — Heatmaps showing the number of overlapping CDR3 sequences across samples for BCR light chain CDR3s (A) and heavy chain CDR3s (B). (PDF) [file ppat.1014094.s004.pdf]

S4 Fig

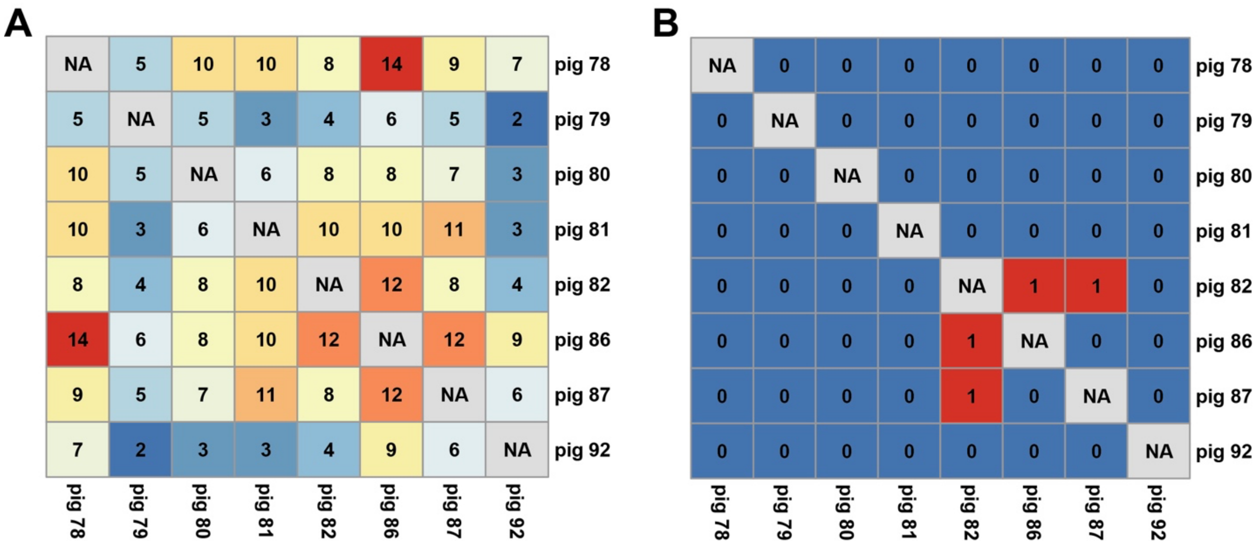
A
NA 5 10 10 8 14 9 7 pig 78
5 NA 5 3 4 6 5 2 pig 79
10 5 NA 6 8 8 7 3 pig 80
10 3 6 NA 10 10 11 3 pig 81
8 4 8 10 NA 12 8 4 pig 82
14 6 8 10 12 NA 12 9 pig 86
9 5 7 11 8 12 NA 6 pig 87
7 2 3 3 4 9 6 NA pig 92
pig 78 pig 79 pig 80 pig 81 pig 82 pig 86 pig 87 pig 92
B
NA 0 0 0 0 0 0 0 pig 78
0 NA 0 0 0 0 0 0 pig 79
0 0 NA 0 0 0 0 0 pig 80
0 0 0 NA 0 0 0 0 pig 81
0 0 0 0 NA 1 1 0 pig 82
0 0 0 0 1 NA 0 0 pig 86
0 0 0 0 1 0 NA 0 pig 87
0 0 0 0 0 0 0 NA pig 92
pig 78 pig 79 pig 80 pig 81 pig 82 pig 86 pig 87 pig 92
