# Supplementary material for: Pigs lacking Natural Killer T cells have altered cellular responses to influenza
Source: PLoS Pathog. 2026 Apr 6;22(4):e1014094. doi: 10.1371/journal.ppat.1014094 (PMC13068344; doi:10.1371/journal.ppat.1014094)
Supplement: S1 Table — (DOCX) [file ppat.1014094.s007.docx]

S1 Table. Frequency (mean ± SEM) of leukocyte populations in lungs at 5 days post challenge

| Immune cell population | Group 1: Vaccinated *CD1D−/−* | Group 2: Vaccinated *CD1D−/+* | Group 3: Unvaccinated *CD1D−/−* | Group 4: Unvaccinated *CD1D−/+* | Group 5: Negative *CD1D−/+* ^a^ |
| --- | --- | --- | --- | --- | --- |
| CD3^+^ (of lymphocytes) | 47.3 ± 3.9 | 35.3 ± 4.1 | 29.1 ± 2 | 26.8 ± 1.2 | 21.5 ± 1.7 |
| αβ cells (CD3^+^TCRδ^-^ of lymphocytes) | 35.1 ± 3.3 | 27 ± 2.7 | 16.7 ± 1.3 | 15.7 ± 1.5 | 14.5 ± 1.9 |
| γδ cells (CD3^+^TCRδ^+^ of lymphocytes) | 7.6 ± 1 | 4 ± 0.6 | 8.9 ± 1 | 7.8 ± 0.7 | 5.4 ± 2.3 |
| CD4^-^CD8α^+^ (of CD3^+^) | 48.5 ± 2.9 | 48.6 ± 2.1 | 42.5 ± 4 | 41.2 ± 1.9 | 37 ± 5.6 |
| CD4^+^CD8α^+^ (of CD3^+^) | 24.2 ± 2.1 | 34.1 ± 3.4 | 23.4 ± 2.3 | 23.3 ± 1.4 | 28.5 ± 3.6 |
| CD4^+^CD8α^-^ (of CD3^+^) | 16.3 ± 0.9 | 12.6 ± 1.2 | 13.1 ± 0.7 | 13.9 ± 2.2 | 16.9 ± 2.1 |
| CD8α^+^ CD8β^+^ (of CD3^+^) | 40.8 ± 4.1 | 39.9 ± 1.9 | 23.3 ± 2.9 | 24.6 ± 2 | 20.2 ± 4.3 |
| NK cells (CD8α^+^CD3^-^ of lymphocytes) | 11.1 ± 1.4 | 12.9 ± 1.2 | 15.4 ± 0.8 | 18.1 ± 1.9 | 13 ± 0.9 |
| Macrophages (CD14^+^CD11b^-^CD163^+^ of leukocytes) | 15.8 ± 0.8 | 19.3 ± 1.7 | 20.1 ± 2.9 | 17.1 ± 1.3 | 19.3 ± 1.8 |
| Monocytes (CD14^+^CD11b^-^CD163^-^ of leukocytes) | 13.9 ± 1.5 | 20.7 ± 3.4 | 15.2 ± 1.3 | 19.8 ± 1.3 | 23.1 ± 0.7 |
| Neutrophils (CD14^+^CD16^+^CD163^-^ of leukocytes) | 2.4 ± 0.3 | 3.8 ± 0.6 | 2.1 ± 0.2 | 2.3 ± 0.3 | 3.5 ± 0.2 |

^a^ The lung of negative *CD1D*-/+ pigs were collected at 17 days post vaccination.
