# Supplementary material for: Pigs lacking Natural Killer T cells have altered cellular responses to influenza
Source: PLoS Pathog. 2026 Apr 6;22(4):e1014094. doi: 10.1371/journal.ppat.1014094 (PMC13068344; doi:10.1371/journal.ppat.1014094)
Supplement: S2 Table — (DOCX) [file ppat.1014094.s008.docx]

S2 Table. Frequency (mean ± SEM) of leukocyte populations in tracheobronchial lymph nodes at 5 days post challenge

| Immune cell population | Group 1: Vaccinated *CD1D−/−* | Group 2: Vaccinated *CD1D−/+* | Group 3: Unvaccinated *CD1D−/−* | Group 4: Unvaccinated *CD1D−/+* | Group 5: Negative *CD1D−/+* ^a^ |
| --- | --- | --- | --- | --- | --- |
| CD3^+^ (of lymphocytes) | 56.6 ± 5.1 | 62.9 ± 6.5 | 20.7 ± 0.8 | 30.1 ± 5.7 | 68 ± 6.7 |
| αβ cells (CD3^+^TCRδ^-^ of lymphocytes) | 50.6 ± 4.8 | 60.2 ± 6.7 | 20.4 ± 1 | 28.2 ± 5.1 | 62.2 ± 6.4 |
| γδ cells (CD3^+^TCRδ^+^ of lymphocytes) | 2.5 ± 0.1 | 1.8 ± 0.1 | 1.5 ± 0.2 | 2.1 ± 0.2 | 3.5 ± 0.7 |
| CD4^-^CD8α^+^ (of CD3^+^) | 21.9 ± 1 | 25.5 ± 1.6 | 23.2 ± 1.3 | 26.8 ± 2.5 | 25.3 ± 1.5 |
| CD4^+^CD8α^+^ (of CD3^+^) | 37.8 ± 6.2 | 46.1 ± 4.4 | 28.4 ± 5.5 | 23 ± 2.8 | 28.1 ± 7.2 |
| CD4^+^CD8α^-^ (of CD3^+^) | 36.1 ± 5.9 | 25.7 ± 3.2 | 37.4 ± 5.3 | 38.6 ± 4.9 | 42.1 ± 6.8 |
| CD8α^+^ CD8β^+^ (of CD3^+^) | 20.6 ± 1.1 | 21.9 ± 1.3 | 22.3 ± 2.2 | 23.8 ± 1.7 | 20.1 ± 0.5 |
| NK cells (CD8α^+^CD3^-^ of lymphocytes) | 10.3 ± 1.8 | 16.2 ± 4.2 | 9.5 ± 3.1 | 4.7 ± 1.6 | 9.1 ± 2.6 |
| Macrophages (CD14^+^CD11b^-^CD163^+^ of leukocytes) | 0.2 ± 0 | 0.6 ± 0.1 | 0.4 ± 0.1 | 0.4 ± 0.1 | 0.9 ± 0.2 |
| Monocytes (CD14^+^CD11b^-^CD163^-^ of leukocytes) | 0.6 ± 0.1 | 0.9 ± 0.2 | 1.1 ± 0.2 | 1 ± 0.1 | 1.7 ± 0.4 |
| Neutrophils (CD14^+^CD16^+^CD163^-^ of leukocytes) | 0.2 ± 0 | 0.4 ± 0.1 | 0.2 ± 0 | 0.3 ± 0.1 | 0.9 ± 0.6 |

^a^ The tracheobronchial lymph node of negative *CD1D*-/+ pigs were collected at 17 days post vaccination.
