# Supplementary material for: Pigs lacking Natural Killer T cells have altered cellular responses to influenza
Source: PLoS Pathog. 2026 Apr 6;22(4):e1014094. doi: 10.1371/journal.ppat.1014094 (PMC13068344; doi:10.1371/journal.ppat.1014094)
Supplement: S3 Table — (DOCX) [file ppat.1014094.s009.docx]

S3 Table. Frequency (mean ± SEM) of leukocyte populations in blood at -1 days post vaccination

| Immune cell population | Group 1: Vaccinated *CD1D−/−* | Group 2: Vaccinated *CD1D−/+* | Group 3: Unvaccinated *CD1D−/−* | Group 4: Unvaccinated *CD1D−/+* | Group 5: Negative *CD1D−/+* |
| --- | --- | --- | --- | --- | --- |
| CD3^+^ (of lymphocytes) | 68.6 ± 3.4 | 68.8 ± 3.1 | 71.6 ± 3.1 | 68.1 ± 2.8 | 63.4 ± 2.9 |
| αβ cells (CD3^+^TCRδ^-^ of lymphocytes) | 30.9 ± 3.6 | 30.5 ± 2.4 | 35.4 ± 0.9 | 28.5 ± 1.9 | 28.9 ± 1 |
| γδ cells (CD3^+^TCRδ^+^ of lymphocytes) | 32.8 ± 4.7 | 30.4 ± 1.9 | 26.7 ± 3.4 | 29.2 ± 3.1 | 25.9 ± 5.7 |
| CD4^-^CD8α^+^ (of CD3^+^) | 17.3 ± 2.7 | 17.5 ± 1.1 | 18 ± 0.9 | 17.7 ± 0.9 | 22.1 ± 1.8 |
| CD4^+^CD8α^+^ (of CD3^+^) | 20.6 ± 8.1 | 13.5 ± 1.6 | 13.2 ± 2 | 11.6 ± 1.3 | 12.6 ± 3.4 |
| CD4^+^CD8α^-^ (of CD3^+^) | 32.2 ± 3.9 | 31 ± 2.1 | 39 ± 1.9 | 32.5 ± 2.9 | 33.4 ± 0.7 |
| CD8α^+^ CD8β^+^ (of CD3^+^) | 12.8 ± 2.1 | 11.7 ± 1.4 | 13.6 ± 0.9 | 12.6 ± 0.7 | 15.2 ± 2.1 |
| NK cells (CD8α^+^CD3^-^ of lymphocytes) | 6 ± 1.5 | 5.3 ± 1.8 | 3.3 ± 0.7 | 4.5 ± 0.8 | 5.3 ± 0.9 |
| Macrophages (CD14^+^CD11b^-^CD163^+^ of leukocytes) | 3.1 ± 0.4 | 3.4 ± 0.4 | 3.1 ± 0.4 | 3.1 ± 0.3 | 2 ± 0.3 |
| Monocytes (CD14^+^CD11b^-^CD163^-^ of leukocytes) | 36.5 ± 8.2 | 31.9 ± 5 | 31.4 ± 2.9 | 34.3 ± 2.8 | 47.8 ± 2.8 |
| Neutrophils (CD14^+^CD16^+^CD163^-^ of leukocytes) | 17.8 ± 1.5 | 12.7 ± 1.4 | 14.6 ± 2.9 | 11 ± 1.9 | 14.1 ± 2.3 |
