# Supplementary material for: Pigs lacking Natural Killer T cells have altered cellular responses to influenza
Source: PLoS Pathog. 2026 Apr 6;22(4):e1014094. doi: 10.1371/journal.ppat.1014094 (PMC13068344; doi:10.1371/journal.ppat.1014094)
Supplement: S4 Table — (DOCX) [file ppat.1014094.s010.docx]

S4 Table. Frequency (mean ± SEM) of leukocyte populations in blood at 14 days post vaccination

| Immune cell population | Group 1: Vaccinated *CD1D−/−* | Group 2: Vaccinated *CD1D−/+* | Group 3: Unvaccinated *CD1D−/−* | Group 4: Unvaccinated *CD1D−/+* | Group 5: Negative *CD1D−/+* |
| --- | --- | --- | --- | --- | --- |
| CD3^+^ (of lymphocytes) | 71.9 ± 1.5 | 66.9 ± 4 | 61.9 ± 4.3 | 56.6 ± 3.3 | 51.5 ± 5.4 |
| αβ cells (CD3^+^TCRδ^-^ of lymphocytes) | 24.9 ± 2.4 | 27.3 ± 2.9 | 28 ± 1.5 | 25.9 ± 1.7 | 26.4 ± 7.4 |
| γδ cells (CD3^+^TCRδ^+^ of lymphocytes) | 33.9 ± 3.8 | 31.1 ± 2.1 | 26 ± 3.4 | 22.3 ± 3.4 | 17.4 ± 8.2 |
| CD4^-^CD8α^+^ (of CD3^+^) | 16.4 ± 1.3 | 17.5 ± 1 | 18.7 ± 2.2 | 18.7 ± 1.4 | 19.8 ± 3 |
| CD4^+^CD8α^+^ (of CD3^+^) | 14 ± 0.9 | 15.8 ± 1.3 | 15.5 ± 2.5 | 14.7 ± 2.3 | 23.9 ± 9 |
| CD4^+^CD8α^-^ (of CD3^+^) | 23.5 ± 2.6 | 23.3 ± 2.3 | 29.2 ± 1.5 | 29.9 ± 2.9 | 30.2 ± 8.9 |
| CD8α^+^ CD8β^+^ (of CD3^+^) | 11.7 ± 1.3 | 12 ± 1.1 | 13.2 ± 1.5 | 13.1 ± 1.2 | 13.8 ± 3.6 |
| NK cells (CD8α^+^CD3^-^ of lymphocytes) | 11.3 ± 0.9 | 11.4 ± 1.1 | 7.6 ± 1.3 | 10.1 ± 0.8 | 13.7 ± 1.7 |
| Macrophages (CD14^+^CD11b^-^CD163^+^ of leukocytes) | 2.3 ± 0.4 | 3 ± 1 | 3.3 ± 0.4 | 2.6 ± 0.9 | 3.6 ± 1 |
| Monocytes (CD14^+^CD11b^-^CD163^-^ of leukocytes) | 27 ± 2.6 | 27.6 ± 2.4 | 34.2 ± 4 | 36.9 ± 3.1 | 48.5 ± 9.3 |
| Neutrophils (CD14^+^CD16^+^CD163^-^ of leukocytes) | 10.5 ± 1.2 | 7.9 ± 0.9 | 12 ± 1.4 | 8.4 ± 1 | 9.4 ± 1.2 |
