# Supplementary material for: Pigs lacking Natural Killer T cells have altered cellular responses to influenza
Source: PLoS Pathog. 2026 Apr 6;22(4):e1014094. doi: 10.1371/journal.ppat.1014094 (PMC13068344; doi:10.1371/journal.ppat.1014094)
Supplement: S5 Table — (DOCX) [file ppat.1014094.s011.docx]

S5 Table. Frequency (mean ± SEM) of leukocyte populations in blood at 20 days post vaccination

| Immune cell population | Group 1: Vaccinated *CD1D−/−* | Group 2: Vaccinated *CD1D−/+* | Group 3: Unvaccinated *CD1D−/−* | Group 4: Unvaccinated *CD1D−/+* | Group 5: Negative *CD1D−/+* ^a^ |
| --- | --- | --- | --- | --- | --- |
| CD3^+^ (of lymphocytes) | 56.3 ± 4.8 | 53.3 ± 4.1 | 63.5 ± 2.8 | 56.9 ± 1.9 | 68.9 ± 4.5 |
| αβ cells (CD3^+^TCRδ^-^ of lymphocytes) | 27.1 ± 2 | 31.1 ± 2.6 | 35.6 ± 2.2 | 34 ± 5.4 | 37.9 ± 3.8 |
| γδ cells (CD3^+^TCRδ^+^ of lymphocytes) | 26.4 ± 3.3 | 15.8 ± 2.4 | 21.1 ± 2.8 | 19.7 ± 3.4 | 18.7 ± 9.7 |
| CD4^-^CD8α^+^ (of CD3^+^) | 16.8 ± 1.4 | 17.3 ± 1.9 | 16.7 ± 1.6 | 18.6 ± 2.1 | 22.2 ± 4 |
| CD4^+^CD8α^+^ (of CD3^+^) | 12.6 ± 1.4 | 14 ± 1 | 13.1 ± 2.1 | 11.1 ± 1.3 | 12 ± 1.5 |
| CD4^+^CD8α^-^ (of CD3^+^) | 30.1 ± 2.1 | 37.4 ± 3.6 | 39.2 ± 1.4 | 38.5 ± 6.4 | 38.6 ± 7 |
| CD8α^+^ CD8β^+^ (of CD3^+^) | 14.5 ± 1.3 | 14.4 ± 1.9 | 14.2 ± 1.4 | 15.3 ± 1.8 | 18.6 ± 4.3 |
| NK cells (CD8α^+^CD3^-^ of lymphocytes) | 6.5 ± 1.9 | 8.1 ± 2 | 5.7 ± 0.8 | 8 ± 1.8 | 2.7 ± 0.4 |
| Macrophages (CD14^+^CD11b^-^CD163^+^ of leukocytes) | 2.2 ± 0.2 | 2.9 ± 0.6 | 1.7 ± 0.3 | 1.8 ± 0.2 | 3 ± 0.6 |
| Monocytes (CD14^+^CD11b^-^CD163^-^ of leukocytes) | 27.1 ± 3 | 29.2 ± 6.5 | 34.5 ± 3.8 | 39.3 ± 4.4 | 47.7 ± 0.5 |
| Neutrophils (CD14^+^CD16^+^CD163^-^ of leukocytes) | 9.2 ± 1.3 | 9.6 ± 1.1 | 12.5 ± 1.5 | 11.1 ± 2.1 | 9.2 ± 1.8 |

^a^ The blood of negative *CD1D*-/+ pigs were collected at 17 days post vaccination.
