# Supplementary material for: Pigs lacking Natural Killer T cells have altered cellular responses to influenza
Source: PLoS Pathog. 2026 Apr 6;22(4):e1014094. doi: 10.1371/journal.ppat.1014094 (PMC13068344; doi:10.1371/journal.ppat.1014094)
Supplement: S6 Table — (DOCX) [file ppat.1014094.s012.docx]

S6 Table. Frequency (mean ± SEM) of leukocyte populations in blood at 5 days post challenge

| Immune cell population | Group 1: Vaccinated *CD1D−/−* | Group 2: Vaccinated *CD1D−/+* | Group 3: Unvaccinated *CD1D−/−* | Group 4: Unvaccinated *CD1D−/+* | Group 5: Negative *CD1D−/+* ^a^ |
| --- | --- | --- | --- | --- | --- |
| CD3^+^ (of lymphocytes) | 70 ± 5.4 | 69.2 ± 3.7 | 71.8 ± 4.9 | 71.7 ± 1.2 | 68.9 ± 4.5 |
| αβ cells (CD3^+^TCRδ^-^ of lymphocytes) | 42.7 ± 1.7 | 51.5 ± 4 | 36.7 ± 2.8 | 39.1 ± 3.8 | 37.9 ± 3.8 |
| γδ cells (CD3^+^TCRδ^+^ of lymphocytes) | 24.2 ± 3.2 | 12.2 ± 3.7 | 31.7 ± 3.5 | 31.3 ± 3.9 | 18.7 ± 9.7 |
| CD4^-^CD8α^+^ (of CD3^+^) | 20.9 ± 1.1 | 24.2 ± 2.9 | 17.4 ± 2.2 | 18.2 ± 1.3 | 22.2 ± 4 |
| CD4^+^CD8α^+^ (of CD3^+^) | 12.7 ± 1.7 | 17.3 ± 2.4 | 9.1 ± 1.3 | 8.5 ± 1.1 | 12 ± 1.5 |
| CD4^+^CD8α^-^ (of CD3^+^) | 34.5 ± 2.4 | 43.4 ± 5.9 | 28.2 ± 3.2 | 31.5 ± 4.8 | 38.6 ± 7 |
| CD8α^+^ CD8β^+^ (of CD3^+^) | 17.7 ± 1.1 | 20.4 ± 2.3 | 15.8 ± 2.8 | 14.9 ± 1.6 | 18.6 ± 4.3 |
| NK cells (CD8α^+^CD3^-^ of lymphocytes) | 2.6 ± 0.6 | 2.8 ± 0.6 | 2.4 ± 1.2 | 2.2 ± 0.3 | 2.7 ± 0.4 |
| Macrophages (CD14^+^CD11b^-^CD163^+^ of leukocytes) | 2.8 ± 0.3 | 4.3 ± 0.9 | 5.8 ± 1.6 | 5.5 ± 1.3 | 3 ± 0.6 |
| Monocytes (CD14^+^CD11b^-^CD163^-^ of leukocytes) | 33.7 ± 3.2 | 46.8 ± 4.5 | 28.1 ± 3 | 36.3 ± 3.1 | 47.7 ± 0.5 |
| Neutrophils (CD14^+^CD16^+^CD163^-^ of leukocytes) | 12.3 ± 2.5 | 13.7 ± 1.5 | 6.5 ± 0.8 | 10.8 ± 1.9 | 9.2 ± 1.8 |

^a^ The blood of negative *CD1D*-/+ pigs were collected at 17 days post vaccination.
