# Supplementary material for: Pigs lacking Natural Killer T cells have altered cellular responses to influenza
Source: PLoS Pathog. 2026 Apr 6;22(4):e1014094. doi: 10.1371/journal.ppat.1014094 (PMC13068344; doi:10.1371/journal.ppat.1014094)
Supplement: S7 Table — (DOCX) [file ppat.1014094.s013.docx]

| Primer name | Target | 5'-3' Sequence | Accession | Ref. type |
| --- | --- | --- | --- | --- |
| TCRa outer | TRAC | ATCGGTGCTTTTGCTCCAAG | MN086839.1 | mRNA |
| TCRa inner | TRAC | GTGGGCTCCGAGTCTTTTGT | MN086839.1 | mRNA |
| TCRb outer | TRBC | TCAGACAGTAGCTGGAGTCATTGAG | AB079894.1 | DNA |
| TCRb inner | TRBC | TCCGATGGTTCAAACACGGC | AB079894.1 | DNA |
| TCRg inner* | TRGC3/4/6 | TCCAGAAGACAAAGGTATGTTCCA | AB185445.1 | mRNA |
| TCRg inner | TRGC5 | TCAAGAAGACAAAGATGTGTCCCA | BK074883 | DNA |
| TCRd outer | TRDC | CTCCATACTGACCAAGCTTGACGG | AB182371.1 | DNA |
| TCRd inner | TRDC | GACCACGATAGCAGGGTCATAT | AB182371.1 | DNA |
| IgA outer | IGHAC | TGCACTTGGCACTTCAGGAT | AB699688.1 | DNA |
| IgA inner | IGHAC | CAATAACGCCCTCGCGACTA | AB699688.1 | DNA |
| IgG outer | IGHGC | CTGAGGGAGTAGAGCCCTGA | AB699686.1 | DNA |
| IgG inner | IGHGC | GCTCGGGGAAGTAGCTTGAG | AB699686.1 | DNA |
| IgM/D outer | IGH(M/D)C | AAGTACTTGCCGCCTCTCAG | AB699686.1 | DNA |
| IgM/D inner | IGH(M/D)C | GATGTTCTGGCTGCTGACCT | AB699686.1 | DNA |
| Kappa outer | IGKC | GAAGCTTTTGACCAGAGGGGA | KF561240.1 | mRNA |
| Kappa inner | IGKC | TCCAGGATGCCACTGCTTTG | KF561240.1 | mRNA |
| Lambda outer | IGLC | CGTCACTGTCTTCTCCACAATG | M59322.1 | mRNA |
| Lambda inner | IGLC | TCTGTTTCGAGGGCTTGGTG | M59322.1 | mRNA |

S7 Table. Porcine custom primer sets for scTCR/BCRseq

* Adapted from ref (48)
