# Supplementary material for: Pigs lacking Natural Killer T cells have altered cellular responses to influenza
Source: PLoS Pathog. 2026 Apr 6;22(4):e1014094. doi: 10.1371/journal.ppat.1014094 (PMC13068344; doi:10.1371/journal.ppat.1014094)
Supplement: S8 Table — (DOCX) [file ppat.1014094.s014.docx]

S8 Table. Shared expanded CDR3β clones between this study and a prior study

| CDR3β | Number of clones in this study | Number of clones in Ref. (35) |
| --- | --- | --- |
| CASSGTGDIQYF | 2 | 3 |
| CASSNSYNSPLHF | 3 | 2 |
| CASSPGQYDYNF | 3 | 2 |
| CASSRDNSPLHF | 11 | 4 |
| CASSRDRDTDPLYF | 2 | 3 |
| CASSRDRGDTYFF | 2 | 5 |
| CASSRDRGTEVFF | 2 | 3 |
| CASSRDSYDYNF | 5 | 2 |
| CASSSAGQGTEVFF | 6 | 3 |
| CASSSELSQTQYF | 14 | 2 |
| CASSTGGYDYNF | 40 | 3 |
| CASSVGSYNDLHF | 6 | 2 |
| CGARGANTGQLYF | 6 | 2 |
| CGASDEDSYDYNF | 4 | 2 |
| CGASDRASRAAQLYF | 4 | 2 |
